# Dermal Delivery of Constructs Encoding Cre Recombinase to Induce Skin Tumors in *Pten^LoxP/LoxP^*;*Braf^CA/+^* Mice

**DOI:** 10.3390/ijms17122149

**Published:** 2016-12-20

**Authors:** Marcel A. Deken, Ji-Ying Song, Jules Gadiot, Adriaan D. Bins, Paula Kroon, Inge Verbrugge, Christian U. Blank

**Affiliations:** 1 Department of Immunology, Netherlands Cancer Institute, Plesmanlaan 121, 1066 CX Amsterdam, The Netherlands; m.deken@nki.nl (M.A.D.); j.gadiot@nki.nl (J.G.); p.kroon@nki.nl (P.K.); i.verbrugge@nki.nl (I.V.); 2 Department of Experimental Animal Pathology, Netherlands Cancer Institute, Plesmanlaan 121, 1066 CX Amsterdam, The Netherlands; j.song@nki.nl; 3 Division of Medical Oncology, Academic Medical Centre, Meibergdreef 9, 1105 AZ Amsterdam, The Netherlands; adbins@gmail.com; 4 Department of Medical Oncology, Netherlands Cancer Institute, Plesmanlaan 121, 1066 CX Amsterdam, The Netherlands

**Keywords:** dermal delivery, *CreER^T2^*, melanoma

## Abstract

Current genetically-engineered mouse melanoma models are often based on *Tyr::CreER^T2^*-controlled MAPK pathway activation by the BRAF^V600E^ mutation and PI3K pathway activation by loss of PTEN. The major drawback of these models is the occurrence of spontaneous tumors caused by leakiness of the *Tyr::CreER^T2^* system, hampering long-term experiments. To address this problem, we investigated several approaches to optimally provide local delivery of Cre recombinase, including injection of lentiviral particles, DNA tattoo administration and particle-mediated gene transfer, to induce melanomas in *Pten^LoxP/LoxP^*;*Braf^CA/+^* mice lacking the *Tyr::CreER^T2^* allele. We found that dermal delivery of the Cre recombinase gene under the control of a non-specific CAG promoter induced the formation of melanomas, but also keratoacanthoma and squamous cell carcinomas. Delivery of Cre recombinase DNA under the control of melanocyte-specific promoters in *Pten^LoxP/LoxP^*;*Braf^CA/+^* mice resulted in sole melanoma induction. The growth rate and histological features of the induced tumors were similar to 4-hydroxytamoxifen-induced tumors in *Tyr::CreER^T2^*;*Pten^LoxP/LoxP^*;*Braf^CA/+^* mice, while the onset of spontaneous tumors was prevented completely. These novel induction methods will allow long-term experiments in mouse models of skin malignancies.

## 1. Introduction

Treatment of metastatic melanoma has changed dramatically within the last decade. Immunotherapy by T cell checkpoint inhibitors, like antibodies targeting cytotoxic T-lymphocyte-associated protein 4 (CTLA-4) and programmed cell death protein 1 (PD-1)/ programmed death-ligand 1 (PD-L1) and targeted therapy by inhibition of an activated mitogen-activated protein kinase (MAPK) pathway, has shown improved tumor responses and/or overall survival [1,2,3,4,5]. The combination of CTLA-4 and PD-1, as well as combined targeting of V600E mutated rapidly accelerated fibrosarcoma kinase B (BRAF^V600E^) and mitogen-activated protein kinase kinase (MEK) within the MAPK pathway have further improved patients’ outcome [5,6,7]. Nevertheless, a long-term benefit is only found in a proportion of patients [8,9]. Since targeted therapy was shown to increase antigen presentation and T cell infiltration, it could provide a rationale to combine with immunotherapy to increase the proportion of patients with long-term benefit [10]. Although early clinical attempts had failed or did not show an additional benefit [11,12,13], more recent studies have shown the promise of combining targeted therapy with immunotherapy [14]. This raises more than ever the need for preclinical evaluation of the safety, efficacy and mechanisms of action of novel combinations of targeted and immunotherapy in melanoma [10,15,16]. Preclinical evaluation is preferably performed in in vivo models in which the tumor microenvironment resembles at least some features of human melanoma. This includes, e.g., vascularization, extracellular matrix and host immune infiltrates. Commonly-used in vivo melanoma models that have a competent immune system include the inoculation of murine melanoma cells (e.g., B16) in syngeneic C57BL/6 mice or engineered mouse models (GEMMs) that have spontaneous tumor formation. More sophisticated GEMMs rely on tumor induction by ultraviolet radiation (UVR), application of 7,12-dimethylbenz(*a*)anthracene (DMBA), viral delivery of RNAs (RCAS/TVA system) or the application of 4-hydroxytamoxifen (Cre/LoxP system) to the skin for melanoma induction [17]. The use of GEMMs allows the study of melanoma in an orthotopic location where the histology and microenvironment has a high resemblance with the human disease. We have previously described a melanoma model harboring the 4-hydroxytamoxifen inducible BRAF^V600E^ mutation and loss of PTEN, under the control of *Tyr::CreER^T2^* [18], a model similar to one described elsewhere [19]. However, these mouse models face the challenge of spontaneous tumor development, due to leaky transport of the CreER^T*2*^ fusion protein to the nucleus [18,20]. This leads to extensive breeding due to early drop-out of animals, causing ethical issues, experimental variation and hindrance of long-term experiments. 

We therefore addressed whether local delivery of Cre recombinase-expressing constructs could overcome this challenge. We found that local delivery of the Cre recombinase gene under the control of the non-specific CMV early enhancer/chicken beta actin (CAG) promoter resulted in the induction of melanomas identical to the 4-hydroxytamoxifen-induced tumors in *Tyr::CreER^T2^*;*Pten^LoxP/LoxP^*;*Braf^CA/+^* mice. However, other non-melanoma skin cancers, such as keratoacanthoma and squamous cell carcinoma, were also induced. Specifically targeting melanocytes by using the tyrosinase promoter resulted in the sole formation of melanomas, which were responsive to selective BRAF inhibition. 

## 2. Results

### 2.1. Spontaneous Tumors Occur in Tyr::CreER^T2^;Pten^LoxP/LoxP^;Braf^CA/+^ Mice

We have previously reported that spontaneous tumors occur in the *Tyr::CreER^T2^*;*Pten^LoxP/LoxP^*;*Braf^CA/+^* mouse melanoma model in the absence of 4-hydroxytamoxifen exposure. These tumors showed rearrangement of the BRAF^V600E^ mutation, loss of phosphatase and tensin homolog (PTEN) and exhibit the same histopathology as the 4-hydroxytamoxifen-induced melanomas [18]. 

**Figure 1:**
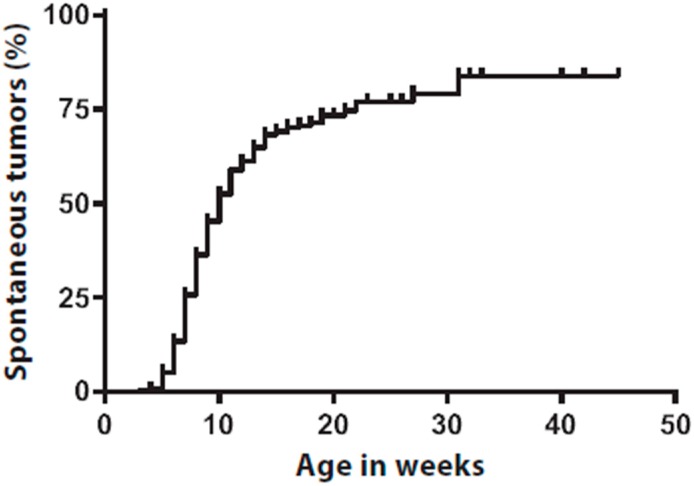
*Tyr::CreER^T2^*;*Pten^LoxP/LoxP^*;*Braf^CA/+^* mice develop spontaneous tumors in the absence of topical 4-hydroxytamoxifen application. Kaplan–Meier analysis showing cumulative occurrence of spontaneous tumors in *Tyr::CreER^T2^*;*Pten^LoxP/LoxP^*;*Braf^CA/+^* mice on body areas where no 4-hydroxytamoxifen was applied. Spontaneous tumors were scored on visual or palpable observation. In 258/379 mice, there was an event of a spontaneous tumor, accounting for 68% of the mice. Spontaneous tumor onset was observed as early as three weeks of age with a median of 10 weeks. Censored survival is indicated by vertical marks in the Kaplan–Meier analysis.

We have now studied in more detail the frequency of such spontaneous tumors. In 258/379 mice, there was an event of a spontaneous tumor, accounting for 68% of the mice. Spontaneous tumor onset was observed as early as three weeks of age with a median of 10 weeks (Figure 1). The spontaneous tumors appeared randomly on the skin, covering the full body of the mice, including areas where they potentially hamper normal behavior and cause substantial discomfort for the animals, e.g., when occurring in close proximity of the limbs or on the facial area. These mice must either be removed from ongoing experiments or are ineligible for inclusion in experiments, hampering a proper experimental setup. 

We previously hypothesized that the occurrence of these spontaneous tumors was most likely due to the leakiness of the inducible Cre system [18]. Absence of *Tyr::CreER^T2^* in the mice completely abolished the onset of spontaneous tumors. Crossing the *Pten^LoxP/LoxP^*;*Braf^CA/+^* mice to another *Tyr::CreER^T2^* mouse strain [21] did not prevent the development of spontaneous tumors (data not shown). These experiments proved that the onset of spontaneous tumors is a result of the leakiness of Cre. We therefore set out to overcome the challenge of disseminated spontaneous onset of tumors. 

### 2.2. Dermal Delivery of CAG-Cre Recombinase DNA Led to Tumor Formation

In our search for a novel tumor induction technique, we aimed to deliver Cre recombinase DNA locally to the skin of mice. We used the intradermal injection of lentiviral particles containing Cre recombinase DNA-expressing vectors, as well as the direct delivery of constructs encoding the Cre recombinase DNA by tattoo administration or particle-mediated gene transfer. 

**Figure 2:**
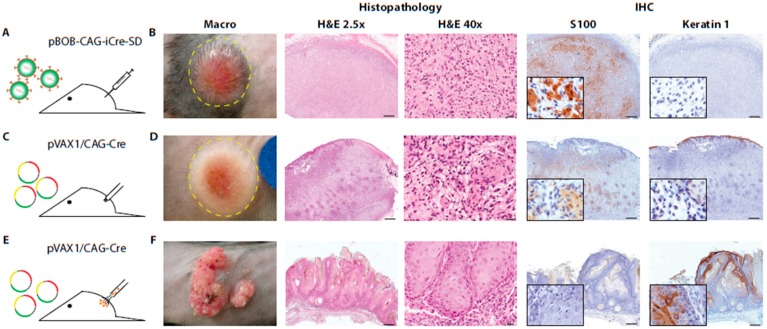
Dermal delivery of Cre recombinase-expressing constructs under the control of the non-specific CAG promoter in *Pten^LoxP/LoxP^*;*Braf^CA/+^* mice led to tumor formation. Tumors were induced in 4–10-week-old *Pten^LoxP/LoxP^*;*Braf^CA/+^* mice by intradermal injection of lentiviral particles containing Cre recombinase DNA-expressing vectors or the direct delivery of constructs encoding the Cre recombinase DNA by tattoo administration or particle-mediated gene transfer, according to the protocols described under the Materials and Methods. (**A**) Schematic representation of intradermal injection of lentiviral particles; (**B**) Macroscopic image and microphotographs of H&E and S100 and keratin 1 staining of a representative Schwannoma-like tumor; (**C**) Schematic representation of DNA tattoo administration; (**D**) Macroscopic picture and H&E and S100 and keratin 1 staining of a representative Schwannoma-like tumor; (**E**) Schematic representation of particle-mediated gene transfer; and (**F**) Macroscopic picture and H&E and S100 and keratin 1 staining of a representative atypical keratoacanthoma. Scale bars: 2.5×: 500 µm; 40×: 20 µm (**B**,**D**,**F**).

Intradermal injection of lentiviral particles containing Cre recombinase in *Pten^LoxP/LoxP^*;*Braf^CA/+^* mice (Figure 2A) resulted in the growth of neoplastic lesions in three out of six mice. Neoplastic lesions were situated subcutaneously, with expansion to the dermis, and the covering epidermis showed local erosive changes. The tumors were rather well demarcated. They were non-pigmented, and the tumor cells were small and round or oval in shape, either loosely present in an edematous background or organized in short bundle structures (Figure 2B). This resembles the Schwannoma-like phenotype of 4-hydroxytamoxifen-induced lesions in *Tyr::CreER^T2^*;*Pten^LoxP/LoxP^*;*Braf^CA/+^* mice [18] and (Figure S1). The tumors showed strong expression of S100, indicating a neural crest origin, but were negative for an epidermal squamous cell marker cytokeratin (Figure 2B). Inflammatory infiltrations of mainly eosinophils were richly present throughout the entire tumor (Figure 2B), which was not seen in 4-hydroxytamoxifen-induced tumors [18] and (Figure S1). Although we observed a reasonable induction of tumors by the injection of lentiviral particles, this approach requires the production of viral particles and handling of infected mice with considerable biosafety requirements. This hampers a high throughput experimental setup and committed us to search for alternative dermal delivery techniques. We therefore investigated the potential of directly delivering naked Cre recombinase DNA to the skin by mechanical means. To test this, naked Cre recombinase encoding plasmids, either without a promoter (pVAX1/noCMV-Cre) or under the control of the non-specific CAG promoter (pVAX1/CAG-Cre), were delivered in vivo in *Pten^LoxP/LoxP^*;*Braf^CA/+^* mice by utilizing a permanent make-up tattoo machine (Figure 2C). This technique, referred to as DNA tattoo administration, results in local transfection of skin cells [22]. DNA tattoo administration of the pVAX1/CAG-Cre construct efficiently induced tumors in the skin. Control DNA tattoo administration with pVAX1/noCMV-Cre did not result in any tumor formation, excluding tumor induction by the DNA tattoo administration itself. Tumor onset was observed in all mice (12/12; 100%; Table 1). Histologically, the lesions were similar to the ones induced by lentiviral delivery of the CAG-Cre construct as described above (Figure 2D), except for the presence of an inflammatory infiltrate. In addition, some tumors showed local pigmentations with nevus-like appearance where the presence of melanocytes and melanophages was evident (Figure S2A). The pigmented lesions were mainly located at the surface of the tumors (Figure S2A). Although inflammatory infiltrations were generally limited, they were increased when erosion of the epidermis took place. In addition, in this cohort, we observed secondary lesions originating from epidermal squamous cells, namely changes like acanthosis, epidermal inclusion cysts, acanthoma, keratoacanthoma, squamous cell papilloma and squamous cell carcinoma. This epidermal squamous cell origin was confirmed by positive immunohistochemical staining for keratin 1, while being negative for S100 (Figure S2B–E). Some of the lesions showed local invasive growth patterns. These lesions either occurred adjacent to the induced tumor or grew as a solitary neoplasm (Figure S1B–E). 

In order to evaluate whether another delivery method resulted in an increased targeting of melanocytes, particle-mediated gene delivery by gene gun was tested as an alternative for the delivery of Cre recombinase constructs (Figure 2E). Mice that were shot with pVAX1/CAG-Cre-coated gold particles developed neoplasms with pigmented nevi-like areas, in a circular projection of the particle-mediated gene transfer (Figure 2F). Histological analysis showed that only neoplastic lesions originating from epidermal squamous cells were encountered, namely the atypical keratoacanthomas, which were positive for keratin 1, while being negative for S100 (Figure 2F). Induction of tumors showing characteristics of Schwann cell-like differentiation, as seen with DNA tattoo administered induction, was not observed. To further increase the likelihood of hitting melanocytes and overcoming the occurrence of secondary tumors originating from non-melanocytic origin, we considered targeting the skin on the ears of mice. It has been shown that the skin of the ears has interfollicular melanocytes instead of the intrafollicular localization found on hairy skin [23], suggestive of a possibly higher efficiency for melanocyte targeting. Particle-mediated delivery of the constructs encoding the Cre recombinase gene again resulted in the induction of tumors with characteristics of Schwann cell-like differentiation, including pigmented lesions (Figure S2F). However, lesions originating from epidermal squamous cells were still frequently observed (Figure S2G and Table 1). 

### 2.3. Dermal Delivery of Cre under the Control of Melanocyte Specific Promoters Results in Melanoma Induction

**Figure 3:**
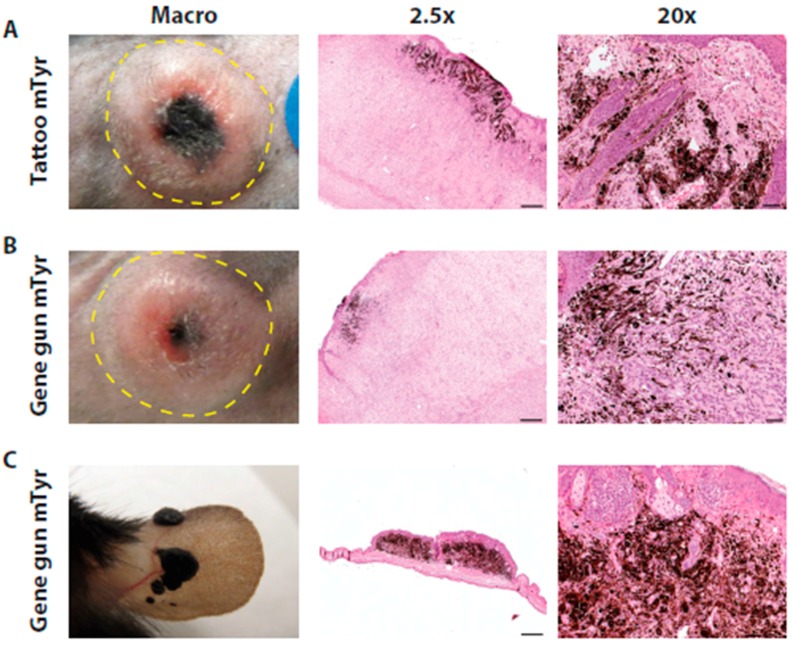
Dermal delivery of Cre recombinase expressing constructs under the control of the melanocyte-specific mTyr promoter in *Pten*^LoxP/LoxP^;*Braf^CA/+^* mice led to tumor formation. Tumors were induced in 4–10-week-old *Pten*^LoxP/LoxP^;*Braf^CA/+^* mice by direct delivery of constructs encoding the Cre recombinase DNA by tattoo administration or particle-mediated gene transfer. (**A**) Macroscopic image and microphotographs of H&E staining of a representative Schwannoma-like tumor with pigmentation induced by DNA tattoo administration on the flank; (**B**) Macroscopic picture and H&E staining of a representative Schwannoma-like tumor with pigmentation induced by particle-mediated gene transfer on the flank; and (**C**) Macroscopic picture and H&E staining of a representative Schwannoma-like tumor with pigmentation induced by particle-mediated gene transfer on the ear. Scale bars: 2.5×: 500 µm; 20×: 50 µm.

The previous experiments showed that local dermal delivery by DNA tattoo administration and particle-mediated gene delivery of constructs encoding the Cre recombinase gene allowed tumor induction. However, the unspecific Cre recombinase expression controlled by the non-specific CAG promoter resulted in the induction of non-melanoma skin cancers. Therefore, we evaluated the expression of the Cre recombinase gene under the control of the melanocyte-specific tyrosinase (Tyr) promoter to allow the specific induction of melanomas. We expected that this specific promoter approach also would overcome the onset of keratoacanthomas and squamous cell carcinomas, which occurred when the Cre recombinase expression was under the control of a non-specific CAG promoter (Figure 2E–F and Figure S1A–C). We therefore generated melanocyte-specific constructs containing the Cre recombinase gene under the control of two versions of the tyrosinase enhancer/promoter region (pVAX1/Tyr-Cre and pVAX1/mTyr-Cre). We considered that relatively short promoters would be able to most efficiently induce tumors, as the size of the original construct might hamper the transfection and translation efficiencies. We therefore generated a minimal tyrosinase promoter (pVAX1/mTyr-Cre) that only harbors the last 500 bp of the 6.1-kb tyrosinase enhancer/promoter region used for pVAX1/Tyr-Cre construct. The size of the original construct might namely hamper the transfection and translation efficiencies. Indeed, utilizing the DNA tattoo administration strategy with the larger plasmids containing the specific promoters, we found that induction of melanomas with pVAX1/Tyr-Cre was less efficient (2/8) as compared to DNA tattoo administration with the pVAX1/mTyr-Cre construct (6/8) (Table 1). Histological analysis of pVAX1/mTyr-Cre DNA tattoo administration-induced tumors showed similar characteristics as previously described for the 4-hydroxytamoxifen inducible mouse model [18], including characteristics of Schwann cell-like differentiation, with the presence of several clusters of melanocytes and melanophages at the surface of the tumor (Figure 3A). Tumors were similarly positive for S100 and negative for keratin 1 immunohistochemical staining (data not shown). Although the epidermal squamous cells showed no neoplastic transformations, the basosquamous cells at the infundibular region of the skin showed hyperplastic changes. In some cases, the presence of nevus-like lesions was found mainly at the surface of the tumors. Inflammatory infiltrations defined by CD3^+^ lymphocytes and F4/80^+^ macrophages were only focally present (Figure S3), resembling the infiltrates of the 4-hydroxytamoxifen inducible mouse model (Figure S3). In vivo particle-mediated gene gun delivery of the melanocyte-specific tyrosinase promoter containing plasmids pVAX1/Tyr-Cre and pVAX1/mTyr-Cre showed induction of melanomas (Tyr and mTyr 2/8), although with a lower efficiency than DNA tattoo administration (Table 1). Histological analysis of pVAX1/mTyr-Cre particle-mediated gene delivery-induced tumors showed similar characteristics as DNA tattoo administration-induced and pVAX1/CAG-Cre and 4-hydroxytamoxifen-induced tumors, including nevi-like pigmented lesions (Figure 3B). To overcome the lower efficiency of induction with the melanocyte-specific tyrosinase promoters, we also targeted the skin on the ears of mice, hoping for an increased likelihood to hit melanocytes [23]. Indeed, induction of neoplasms was observed on the ears with a higher efficiency (Tyr-Cre 2/4 and mTyr-Cre 4/4, respectively) than on the flank skin (Tyr-Cre and mTyr-Cre; 2/8). These tumors were highly pigmented, and histological analysis revealed a melanoma phenotype that had similar characteristics to the tumors described above, namely a Schwann cell-like differentiation, but with massive pigmentations in the tumor (Figure 3C). The induced melanomas on the ears showed a slower growth progression (Figure S4).

### 2.4. Tumors Induced by Dermal Delivery Were Sensitive to Selective BRAF Inhibition

**Figure 4:**
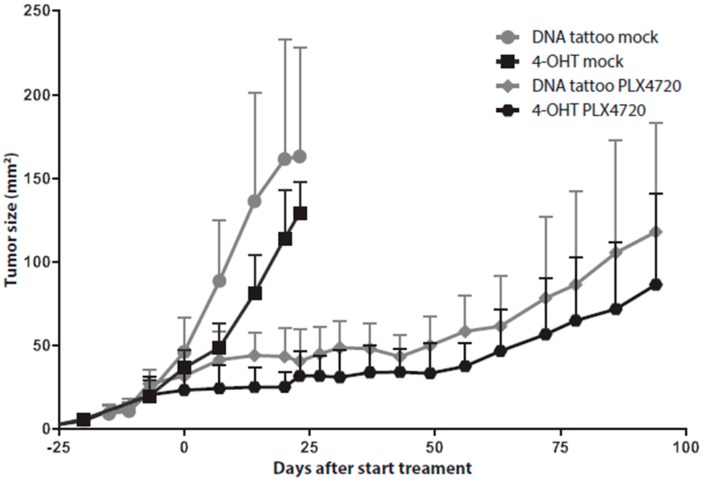
Inhibition of BRAF^V600E^ in induced tumors led to tumor outgrowth inhibition. Melanomas were induced by 4-hydroxytamoxifen application on the flank of 4–10-week-old C57BL/6J *Tyr::CreER^T2^*;*Pten^LoxP/LoxP^*;*Braf^CA/+^* mice or DNA tattoo administration of the pVAX1/mTyr-Cre construct on the flank of *Pten^LoxP/LoxP^*;*Braf^CA/+^* mice (*n* = 5 per group). When the average tumor size was 25 mm^2^, tumor-bearing mice were placed on PLX4720 chow. Tumor outgrowth was monitored over time by digital photography, and tumor size was plotted against time from tumor induction for PLX4720-treated and control chow mock-treated animals. Data are expressed as the means ± SD.

To investigate the proliferative capacity of the alternatively-induced tumors, we compared their growth rate with that of the 4-hydroxytamoxifen-induced tumors in *Tyr::CreER^T2^*;*Pten^LoxP/LoxP^*;*Braf^CA/+^* mice. Tumors in *Pten^LoxP/LoxP^*;*Braf^CA/+^* mice induced by pVAX1/mTyr-Cre DNA tattoo administration showed the same growth rate compared to 4-hydroxytamoxifen-induced tumors in *Tyr::CreER^T2^*;*Pten^LoxP/LoxP^*;*Braf^CA/+^* mice (Figure 4). We have shown previously that selective inhibition of Braf^V600E^ with PLX4720 in *Tyr::CreER^T2^*;*Pten^LoxP/LoxP^*;*Braf^CA/+^* mice led to a growth inhibition of the 4-hydroxytamoxifen-induced tumors [18]. DNA tattoo administration-induced tumors were equally susceptible to BRAF inhibition by PLX4720 (Figure 4), again confirming MAPK pathway activation as the oncogenic driver in these tumors. 

## 3. Discussion

Targeted and immunotherapy of late-stage melanoma have significantly improved patients’ outcome. Targeting BRAF^V600E^ and MEK and immunotherapies, such as CTLA-4 and PD-1 immune checkpoint blockade, have become first-line treatment options. However, long-term benefit can be expected for less than 50% of patients [8,9]. These observations have led to the idea of combining targeted- and immunotherapy to further improve response rates and long-term outcome, with some combinations already in clinical testing [10,24]. To gain further insight into promising combination therapies and to test different on/off combination schemes (e.g., synchronous or sequential combination therapies), preclinical in vivo models are highly needed. In the work described here, we tested different tumor induction methods in a mouse melanoma model based on the 4-hydroxytamoxifen-based tumor induction of *Tyr::CreER^T2^*;*Pten^LoxP/LoxP^*;*Braf^CA/+^* mice [18,19]. A major caveat of this original model is the occurrence of spontaneous tumors caused by the leakiness of the *Tyr::CreER^T2^* allele [18,20]. The novel induction methods we have established here enable the testing of combination therapies in long-term experiments without the occurrence of spontaneous tumors.

The induction of tumors by the injection of lentiviral particles containing Cre recombinase-expressing DNA resulted in the growth of tumors that phenotypically resembled the 4-hydroxytamoxifen-induced tumors in the *Tyr::CreER^T2^*;*Pten^LoxP/LoxP^*;*Braf^CA/+^* mice [18]. However, these tumors demonstrated relatively strong inflammatory infiltrations defined by the presence of eosinophils, which might be due to the viral nature of this induction method. Another challenge for utilizing this lentiviral approach is the intensive safety regulations for the handling of virus particles and infected animals. Our work has identified dermal delivery of DNA constructs encoding the Cre recombinase gene to the skin by DNA tattoo administration and particle-mediated gene delivery as superior approaches for tumor induction. DNA tattoo administration of constructs expressing the Cre recombinase gene under the control of the non-specific CAG promoter resulted in the growth of tumors that were more similar to that of 4-hydroxytamoxifen-induced tumors in *Tyr::CreER^T2^*;*Pten^LoxP/LoxP^*;*Braf^CA/+^* mice. However, both the DNA tattoo administration and the particle-mediated gene delivery approach also resulted in the occurrence of keratoacanthoma and squamous cell carcinoma, due to the absence of melanocyte-specific targeting. The development of keratoacanthoma and cutaneous squamous-cell carcinomas in melanoma patients treated with the selective BRAF inhibitor vemurafenib has been linked to the presence of frequent mutations in RAS, particularly HRAS [25]. The molecular mechanism is consistent with the paradoxical activation of MAPK signaling and results in the accelerated growth of these lesions [25]. Our data show, for the first time, the induction of keratoacanthoma and squamous cell carcinoma on a background of BRAF^V600E^ and loss of PTEN. Using a keratinocyte-specific promoter, such as the bovine keratin (K)5 or involucrin promoters [26,27], would allow the generation of novel inducible mouse models that could be of interest for studies on keratoacanthoma and cutaneous squamous cell carcinoma development. 

To further increase the likelihood of hitting melanocytes and to overcome the occurrence of secondary tumors originating from non-melanocytic origin, we targeted the skin on the ears of mice. It has been shown that in the skin of the ears, melanocytes have a predominant interfollicular localization [23,28]. Due to their interfollicular presence in the dermis, the melanocytes do not undergo hair-cycle-related regression and deletion. The melanocytes are thus constantly present, and this could lead to a better efficiency of targeting pigmented melanocytes. Indeed, we observed a high tumor induction efficiency when particle-mediated gene delivery was used to deliver the pVAX1/CAG-Cre construct in the ear of *Pten^LoxP/LoxP^*;*Braf^CA/+^* mice. Although all tumors showed pigmented nevi, it did not prevent the occurrence of secondary tumors. To further study the alternatives to prevent the occurrence of secondary tumors, we used melanocyte-specific promotors. The DNA tattoo administration strategy with plasmids containing melanocyte-specific promoters was more effective using a minimally-sized pVAX1/mTyr-Cre construct as compared to the larger pVAX1/Tyr-Cre constructs. This might reflect previous studies showing that transfection of cells and transcription/translation of transfected genes is dependent on the size of the construct [29,30]. Furthermore, the efficiency of tumor induction will be dependent on the respective endogenous promoter activity. Interestingly, tumors that were induced on the ear of *Pten^LoxP/LoxP^*;*Braf^CA/+^* mice by particle-mediated gene transfer with melanocyte-specific tyrosinase promoters resulted in the occurrence of slow progressing pigmented lesions that did not become depigmented. These lesions with strong pigmentation resembled the pigmented melanocytic lesions we previously induced with 4-hydroxytamoxifen in *Tyr::CreER^T2^*;*Pten^LoxP/LoxP^*;*Braf^CA/+^* mice.

The tumor induction efficiencies we observed are likely to correlate with the localization and maturation status of melanocytes, which differs during the different phases of the hair cycle and is specific for a respective anatomic site. Furthermore, specifically in the anagen phase of the hair cycle, hair follicles are accompanied by a large amount of melanocytes [31,32]. These follicular melanocytes are thought to give rise to the tumors in inducible mouse melanoma models [28]. We did not analyze the hair cycle at the moment of induction, but hypothesize that it might be possible to further increase the tumor induction efficiency by inducing the anagen of the hair cycle by depilation. 

Our novel induction method is complementary to the RCAS/TVA system with respect to overcoming the occurrence of spontaneous tumors [33,34]. The RCAS/TVA system utilizes the subcutaneous injection of RCASBP(A)GFP-infected avian fibroblast DF-1 producer cells or supernatant to induce tumors in neonatal mice harboring the DCT-TVA allele. They achieved a tumor induction efficiency of 36% in *Ink4a/Arf^lox/lox^* mice, whereas tumor development in *Dct::TVA*;*Braf^CA^*;*Cdkn2a^lox/lox^* and *Dct::TVA*;*Braf^CA^*;*Cdkn2a^lox/lox^*;*Pten^Lox/Lox^* mice had an efficiency of 47% and 100%, respectively [33,34]. As has been discussed [17], the utilization of the RCAS/TVA system has limitations, as it requires actively dividing cells, and integration is thought to be random. Not only can this potentially affect the expression of host genes, but also may lead to random immunogenicity, which will be highly unwanted with respect to immunological studies. 

In summary, we have tested different induction methods of melanoma in the *Pten^LoxP/LoxP^*;*Braf^CA/+^* mouse melanoma model. These techniques will also be applicable to other mouse models that rely on local induction of skin malignancies. Utilizing the DNA tattoo administration or particle-mediated gene delivery methods results in efficient induction of melanoma with different outgrowth characteristics of pigmented and non-pigmented melanomatous lesions. To our knowledge, this is the first time dermal delivery of DNA has been used for the induction of tumors. Both particle-mediated gene delivery and DNA tattoo administration induction methods enable the generation of mouse models, including, but not limited to, BRAF^V600E^/PTEN^−/−^ melanoma, suitable for long-term tumor experiments. In the current exciting times of emerging novel therapies, this provides relevant mouse models to study melanoma and other skin cancers. 

## 4. Materials and Methods

### 4.1. Mouse Model and Tumor Induction

The inducible *Tyr::CreER^T2^*;*Pten^LoxP/LoxP^*;*Braf^CA/+^* melanoma model has been described before [18]. We obtained the *Tyr::CreER^T2^*;*Pten^LoxP/LoxP^*;*Braf^CA/+^* and *Pten^LoxP/LoxP^*;*Braf^CA/+^* mice by crossing the C57BL/6J *Tyr::CreER^T2^*, C57BL/6J *Pten^LoxP/LoxP^* and *Braf^CA/+^* mouse strains as previously described [18]. All described animal experiments were approved by the Animal Experimentation Committee of The Netherlands Cancer Institute (research plans 11.031 B24—23 January 2013 and 11.031 B37—19 June 2014). Mice were treated in accordance with the Dutch law on animal experimentation.

### 4.2. Viral Production and Plasmid Construction

The pBOB-CAG-iCRE-SD plasmid (a gift from Inder Verma, Addgene Plasmid #12336) was used for the production of CAG-iCRE containing lentiviral particles. Lentiviral particles were produced in HEK-293 T cells by polyethylenimine transfection with the lentiviral construct and helper plasmids pMDLglpRRE, pHCMV-G and pRSVrev (kindly provided by Daniel Peeper, Netherlands Cancer Institute, Amsterdam, The Netherlands). The medium of the infected HEK-293 T cells was refreshed after 24 h, after which they were allowed to produce viral particles for 24 h. The supernatant was spun down, 0.45 μm filtered and concentrated using an ultracentrifuge. Plasmids for dermal naked DNA delivery were constructed by using the minimal pVAX1 vector (Life Technologies, Carlsbad, CA, USA) as a backbone. pVAX1/CAG-Cre was generated by the insertion of the CAG-Cre sequence from pCAG-Cre (a gift from Connie Cepko, Addgene Plasmid #13775) [35] by restriction with SpeI/NotI, which resulted in the replacement of the CMV promoter previously present in the pVAX1 vector. pVAX1/noCMV-Cre was generated by first removing the CMV promoter in the pVAX1 backbone by *Spe*I digestion and direct re-ligation. Secondly, the Cre recombinase gene was inserted by *xba*I/*Apa*I in the remaining multiple cloning site after amplification by PCR from pCAG-Cre, while introducing the *xb*aI/*Apa*I restriction sites (forward primer, 5′TATCTAGATGAGCCGCCACCATGGCC3′; and reverse primer, 5′TAGGGCCCCTATCACAGATCTTCTTCAGAAA3′). The UPT plasmid that contains the 6.1-kb tyrosinase enhancer/promoter sequence in pBluescript II SK(^+^), originally constructed by Susan Porter [36] (University of British Columbia, Vancouver, BC, Canada), was kindly provided by Lionel Larue (Institute Curie, Paris, France). This sequence is identical to the one used in the *Tyr::CreER^T2^* mouse strain used in our mouse model [18,37]. pVAX1/Tyr-Cre was generated by insertion of the tyrosinase enhancer/promoter sequence from the UPT plasmid into the *Eco*RI restriction site present right before the Cre recombinase sequence of pVAX1/noCMV-Cre. Correct orientation of the insert was confirmed using primers outside the *Eco*RI site (forward primer, 5′GATGTACGGGCCAGATATACGCG3′; and reverse primer, 5′AGTGGGAGTGGCACCTTCCAG3′). pVAX1/mTyr-Cre was generated by insertion of the 577-bp promoter sequence [38] into the *Not*I/*xho*I restriction sites of pVAX1/noCMV-Cre after the sequence was synthesized as a GeneArt DNA fragment (Life Technologies, Carlsbad, CA, USA), introducing *Not*I/*xho*I restriction sites. All PCR and sequencing primers are shown in Table S1.

### 4.3. Tumor Induction

Tumors in *Pten^LoxP/LoxP^*;*Braf^CA/+^* mice were induced by dermal delivery of genes encoding Cre recombinase DNA in the mouse skin by (1) utilizing lentiviral particle injection; (2) particle-mediated gene transfer or (3) DNA tattoo administration. Biolistic gene gun particle-mediated DNA delivery was performed using a self-fabricated airsoft handgun connected to a helium cylinder. Bullets were produced by coating the inner lining of 1/8″ EFTE tubing (Saint-Gobain, Mickelton, NJ, USA) with protamine and precipitated circular plasmid DNA on spherical gold microparticles (Alfa Aesar, Ward Hill, MA, USA). The tubing was cut to fit the cartridge of the gene gun and shot using pressurized helium to accelerate the DNA-coated gold particles into the ear and flank of *Pten^LoxP/LoxP^*;*Braf^CA/+^* mice. Tumor induction by DNA tattooing was performed by utilizing an Intelligence^TM^ permanent make-up machine (Nouveau Contour, The Netherlands), as described previously [22,39,40]. DNA tattoo administration was being performed by applying 5 µL of a 4–5 mg/mL DNA solution to an ~1-cm^2^ area on the shaven right flank of mice, for three consecutive days. The machine was set at a frequency of 100 Hz. The target area for tattooing was marked with a permanent marker pen. Tumor induction on the skin of the *Tyr::CreER^T2^*;*Pten^LoxP/LoxP^*;*Braf^CA/+^* mice was performed as previously described [18]. In short, a droplet of 2 μL of 4-hydroxytamoxifen dissolved in DMSO is topically applied for 5 min to the shaven right flank of mice for three consecutive days. Tumor outgrowth was followed twice weekly by digital photographing of the tumor, including a sticker as a size reference. Tumor size was analyzed using ImageJ software (National Institutes of Health, Bethesda, MA, USA). 

### 4.4. Treatment of Tumor-Bearing Mice

Selective BRAF^V600E^ inhibition was tested by providing chow containing PLX4720, a research analog of vemurafenib (PLX4032), provided by Plexxikon (Berkeley, CA, USA). At the start of the experiments, the mice were switched either to a chow diet containing 417 mg/kg PLX4720 or to control chow. On average, the food dosing is similar to a daily 50 mg/kg dosing by oral gavage [18,41]. 

### 4.5. Histological and Immunohistochemical Analysis

Tissues were removed from the mice directly after euthanasia. They were fixed in formalin and embedded in paraffin. Two micrometer-thick sections were made and stained by hematoxylin and eosin (H&E) according to standard procedures. Four micrometer-thick sections were made and stained with antibodies anti-S100 (DakoCytomation; clone Z0311), keratin 1 (Covance; Clone PRB-165P), F4/80 (Serotec; Clone MCA497) or CD3 (Neomarkers; Clone RM-9107) according to standard immunohistochemistry (IHC) protocols. The sections were reviewed with a Zeiss Axioskop2 Plus microscope (Carl Zeiss Microscopy, Jena, Germany), and images were captured with a Zeiss AxioCam HRc digital camera and processed with AxioVision 4 software (both from Carl ZeissVision, München, Germany). 

## Figures and Tables

**Table 1:** Tumor induction efficiency by dermal delivery of Cre recombinase. Tumors were induced by different dermal delivery techniques and constructs expressing the Cre recombinase gene. The efficiency of tumor induction was scored as neoplastic growth and pathological confirmation. Mean tumor latency and histological characterization are presented.

Delivery Method	Construct	*n*	*%*	Mean Latency	Tumor Type
Intradermal injection	pBOB-CAG-iCRE-SD	3/6	*50*	*73d*	melanoma *
Tattoo flank	pVAX1/noCMV-Cre	0/6	*0*	*-*	
	pVAX1/CAG-Cre	12/12	*100*	*27d*	melanoma * and secondary lesions
	pVAX1/Tyr-Cre	2/8	*25*	*29d*	melanoma *
	pVAX1/mTyr-Cre	6/8	*75*	*42d*	melanoma *
Gene gun flank	pVAX1/CAG-Cre	8/8	*100*	*33d*	*epidermal lesions*
	pVAX1/Tyr-Cre	2/8	*25*	*50d*	melanoma *
	pVAX1/mTyr-Cre	2/8	*25*	*42d*	melanoma *
Gene gun ear	pVAX1/CAG-Cre	4/4	*100*	*56d*	melanoma * and secondary lesions
	pVAX1/Tyr-Cre	2/4	*50*	*59d*	melanoma *
	pVAX1/mTyr-Cre	4/4	*100*	*56d*	melanoma *

* = melanoma with Schwannoma-like characteristics as described in the Results.

## Supplementary Materials

Supplementary materials can be found at www.mdpi.com/1422-0067/17/12/2149/s1.

## Abbreviations

Tyr	Tyrosinase promoter (6.1-kb version)
mTyr	Tyrosinase promoter (577-bp version)
