# Supplementary material for: Dermal Delivery of Constructs Encoding Cre Recombinase to Induce Skin Tumors in PtenLoxP/LoxP;BrafCA/+ Mice
Source: Int J Mol Sci. 2016 Dec 20;17(12):2149. doi: 10.3390/ijms17122149 (PMC5187949; doi:10.3390/ijms17122149)
Supplement: Supplementary file 1 [file ijms-17-02149-s001.pdf]

Supplementary Materials: Dermal Delivery of Constructs Encoding Cre Recombinase to Induce Skin Tumors in *Pten*$^{LoxP/LoxP}$;*Braf*$^{CA/+}$ Mice

Marcel A. Deken, Ji-Ying Song, Jules Gadiot, Adriaan D. Bins, Paula Kroon, Inge Verbrugge and Christian U. Blank

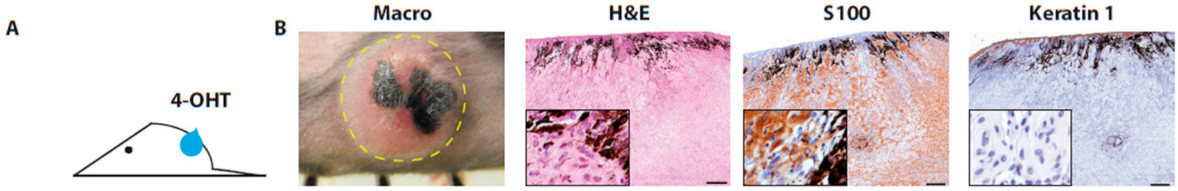

Figure S1. Dermal application of 4-hydroxytamoxifen (4-OHT) to the skin of *Tyr::CreER*^{T2}; *Pten*$^{LoxP/LoxP}$;*Braf*$^{CA/+}$ mice led to tumor formation; (**A**) Schematic representation of 4-hydroxytamoxifen application; (**B**) Macroscopic image and microphotographs of H&E and S100 and keratin 1 staining of a representative tumor. Scale bar: 2.5×: 500 μm; the width of the zoomed insert is 130 μm.

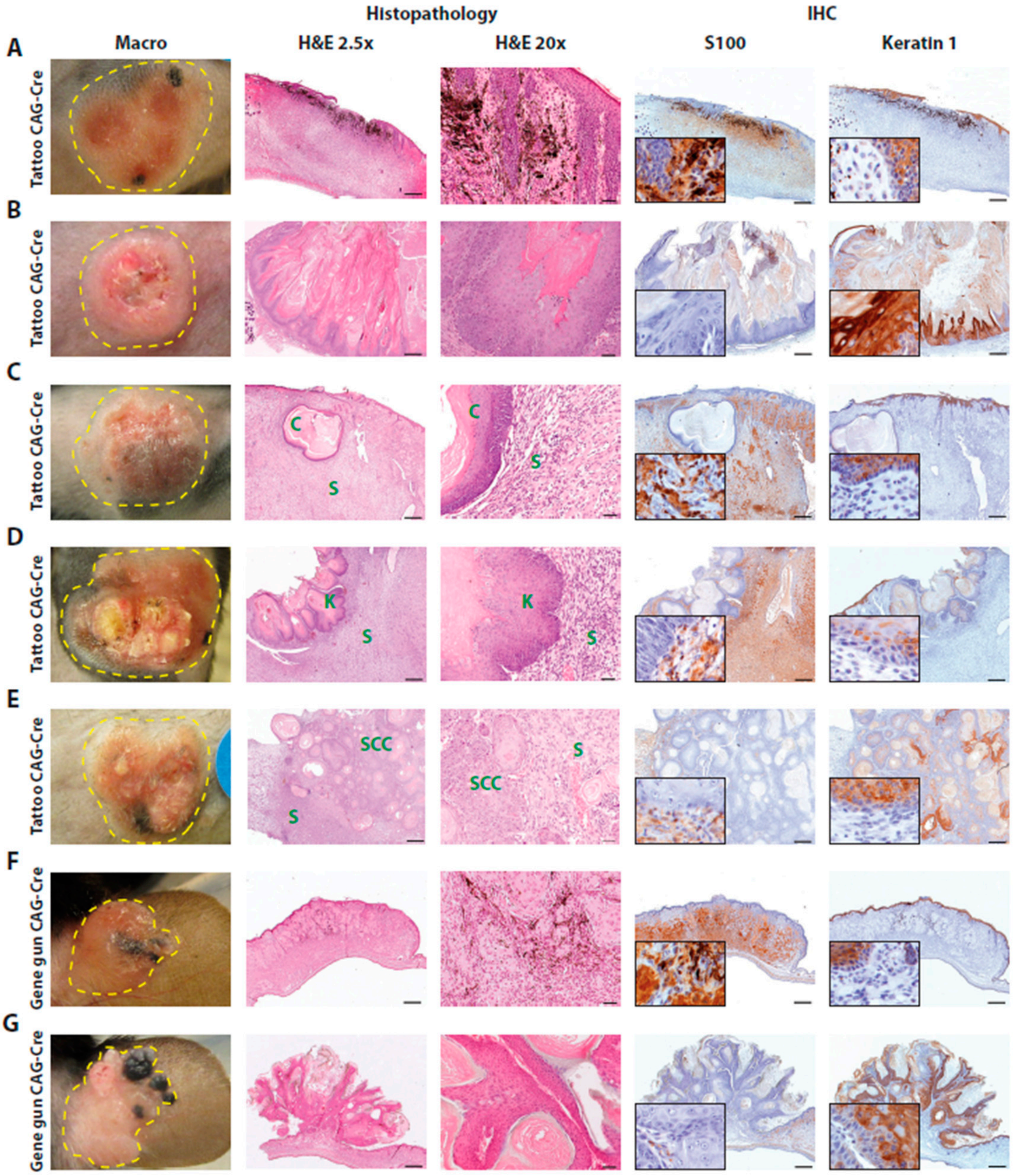

Figure S2. Dermal delivery of Cre recombinase expressing constructs under control of the non-specific CAG promoter or the specific mTyr promoter in *Pten*$^{LoxP/LoxP}$;*Braf*$^{CA/+}$ mice led to tumor formation. Tumors were induced in 4–10-week-old *Pten*$^{LoxP/LoxP}$;*Braf*$^{CA/+}$ mice by the direct delivery of constructs encoding the Cre recombinase DNA by tattoo administration or particle-mediated gene transfer; (**A**–**E**) Macroscopic image and microphotographs of H&E and S100 and keratin 1 immunohistochemical stainings of tumors induced by DNA tattoo administration of pVAX1/CAG-Cre on the flank showing: (**A**) pigmented nevi with Schwannoma like lesions underneath; (**B**) keratoacanthomas; (**C**) lesion with Schwannoma-like characteristics and epidermal inclusion cyst; (**D**) lesion with atypical keratoacanthoma and Schwannoma-like lesions underneath; and (**E**) squamous cell carcinoma mixed with Schwannoma-like characteristics; (**F**,**G**) Macroscopic image and microphotographs of H&E and S100 and keratin 1 stainings of tumors induced by particle-mediated gene transfer of pVAX1/CAG-Cre on the ear showing: (**F**) lesion with pigmented regions, Schwannoma-like characteristics; (**G**) a macroscopically-mixed lesion with pigmented nevi, squamous cell papilloma and keratoacanthomas, microphotographs of H&E and S100 and keratin 1 stainings of squamous cell papilloma. Abbreviations: C, cyst; IHC, immunohistochemistry; K, keratoacanthomas; S, Schwannoma characteristics; SCC, squamous cell carcinoma. Scale bars: 2.5×: 500 μm; 20×: 50 μm; the width of the zoomed insert is 130 μm.

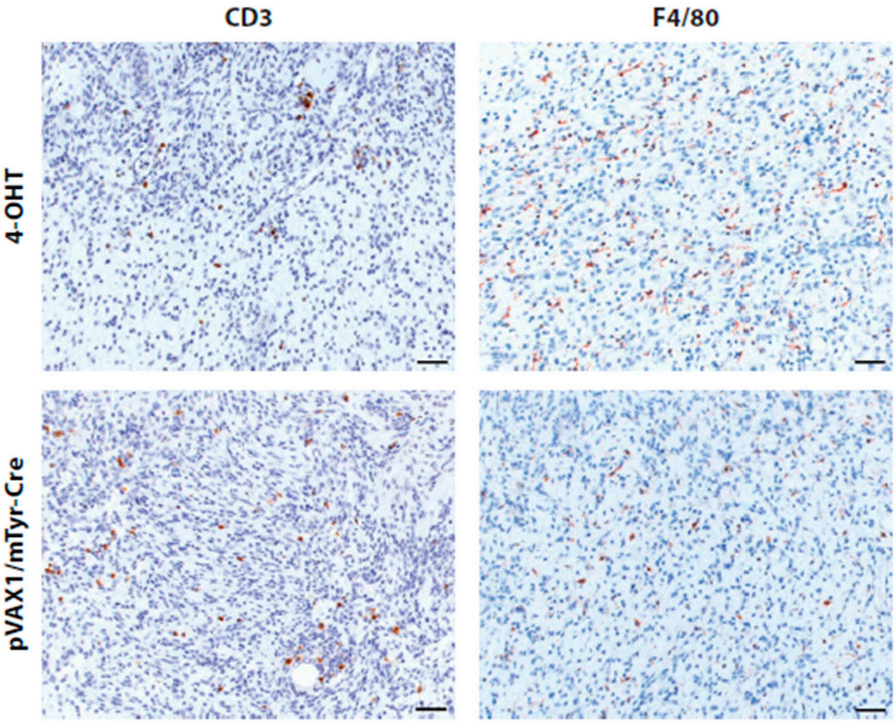

Figure S3. Representative microphotographs of CD3 (T lymphocytes) and F4/80 (macrophages) immunohistochemical stainings of tumors induced by 4-hydroxytamoxifen (4-OHT) in *Tyr::CreER*^{T2};*Pten*$^{LoxP/LoxP}$;*Braf*$^{CA/+}$ mice and induced by DNA tattoo-mediated delivery of pVAX1/mTyr-Cre in *Pten*$^{LoxP/LoxP}$;*Braf*$^{CA/+}$ mice. Scale bar: 2.5×: 500 μm.

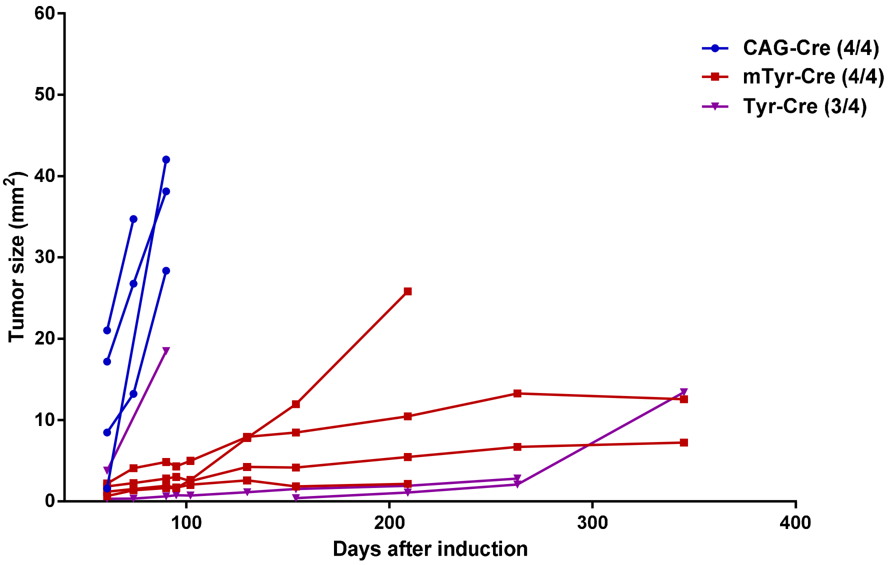

Figure S4. Outgrowth of tumors induced by particle-mediated gene transfer on the ear. Tumors were induced by particle-mediated gene transfer of constructs expressing the Cre recombinase gene on the right ear of 4–10-week-old *Pten*$^{LoxP/LoxP}$;*Braf*$^{CA/+}$ mice. Tumors were induced by the transfer of non-specific construct pVAX1/CAG-Cre (blue lines); or melanocyte-specific constructs pVAX1/mTyr-Cre (red lines); and pVAX1/Tyr-Cre (purple lines). Tumor outgrowth was monitored over time by digital photography, and tumor size was plotted against time from tumor induction. Data are expressed per individual mouse.

Table S1. Primers used to introduce restriction sites or for sequence verification.

Name	Sequence
XbaI-Cre PCR forward	5′TATCTAGATGAGCCGCCACCATGGCC3′
Cre-ApaI PCR reverse	5′TAGGGCCCCTATCACAGATCTTCTTCAGAAA3′
pVAX1-noCMVcheck forward	5′GATGTACGGGCCAGATATACGCG3′
pVAX1-noCMVcheck reverse	5′AGTGGGAGTGGCACCTTCCAG3′
